# Relationship between hair shedding and systemic inflammation in COVID-19 pneumonia 

**DOI:** 10.1080/07853890.2022.2054026

**Published:** 2022-03-26

**Authors:** Gabriella Guarnieri, Leonardo Bertagna De Marchi, Alessandro Marcon, Silvia Panunzi, Veronica Batani, Marco Caminati, Fabiana Furci, Gianenrico Senna, Mauro Alaibac, Andrea Vianello

**Affiliations:** a Department of Cardiac-Thoracic-Vascular Sciences and Public Health, Respiratory Pathophysiology Unit, University of Padova, Padova, Italy; b Department of Diagnostics and Public Health, Unit of Epidemiology and Medical Statistics, University of Verona, Verona, Italy; c Department of Medicine, University of Verona and Verona University Hospital, Verona, Italy; d Allergy Unit and Asthma Center, Verona University Hospital, Verona, Italy; e Department of Medicine-DIMED, Unit of Dermatology, University of Padova, Padova, Italy

**Keywords:** Telogen effluvium, interleukin 1 β levels, androgens, transmembrane protease serin 2, alopecia

## Abstract

**Background:** A higher risk for COVID-19 infection and severity for men compared to women has been described since the beginning of the pandemic. The role of androgens has been recently highlighted as they control two key steps of coronavirus infection mediated through the transmembrane protease serin 2 (TMPRRS2) and the angiotensin-converting enzyme 2 (ACE2) receptor in the lung tissue. Furthermore, a high incidence of androgenic alopecia among males with COVID-19 disease have been reported.

**Objective:** This study aims to evaluate the telogen effluvium (TE) prevalence and its relationship with clinical and immunologic parameters in a sample of patients consecutively evaluated after recovery from COVID-19 pneumonia in Northern Italy.

**Methods:** Overall 104 patients were recruited within three months from COVID-19 pneumonia recovery; 80 (77%) had been hospitalized in a Respiratory Intensive Care Unit and the remaining ones had been treated at home. The extent of TE was assessed with a visual analogic scale for thick bundle of hairs. Demographic and clinical data and systemic inflammation biomarkers were also evaluated.

Results. Thirty-two patients reported a history of TE and their mean TE-VAS score was 5.78 ± 1.72 (range 3–9). Women had about a 5-fold higher risk (odds) of complaining of TE compared to males (OR = 4.69, 95%CI: 1.91, 11.49; *p* = .001), and the association became stronger when adjusted for COVID-19 severity (hospital admission vs home care: OR = 6.09, 95%CI: 2.34, 15.88; *p * < .001). Levels of C-reactive protein >1.90 mg/l (OR_adj_: 2.43, 95%CI 0.85, 7.05, *p* = 0.096) or IL 1β > 5 ng/l (OR_adj_ 4.72, 95%CI: 1.31, 23.19, *p* = .03) were also significantly associated with TE.

**Conclusion:** This exploratory study raises the hypothesis that hair shedding is more strictly related to the severity of COVID-19 disease and the underlying inflammation rather than to patients' hormonal status.

**KEY MESSAGES**
- The presence of Telogen effluvium (TE) was significantly more common in women.
- Higher severity of the Covid-19 disease seems to play a critical role, more important than the hormonal influence, in the development of TE.
- The severity of inflammation related to TE and Covid-19 could also play a role as suggested by the higher levels of CRP and platelets and IL1β.

## Introduction

A higher risk and severity of COVID-19 for men compared to women has been described since the beginning of the pandemic [1]. Several explanations for such gender gap may be advocated, including the higher prevalence of systemic comorbidities such as hypertension, cardiac diseases, diabetes, and obesity among males [2]. Moreover, the role of androgens has been recently highlighted as they control two key steps of coronavirus infection. In fact, the expression of both the transmembrane protease serin 2 (TMPRRS2) and the angiotensin-converting enzyme 2 (ACE2) receptor is regulated in the lung tissue by androgens. Up-regulated TMPRRS2 primes SARS CoV2 spike protein S, enabling it to interact with the ACE2 receptor and to enter the host cell. This hypothesis on the role of androgens in COVID-19 is strengthened by several observations, including the low number of infected patients under the age of 14 years, which could be linked to their physiologically low levels of androgens [3], as well as the detection of an increased incidence of androgenetic alopecia among males with COVID-19 disease [4]. A higher risk for males was also reported for the Middle East respiratory syndrome epidemic [5]. Moreover, alopecia, presence of grey hair, and baldness has been associated with a greater disease severity [6–8]. According to these data, a potential therapeutic role of finasteride or other five alpha-reductase inhibitors in COVID-19 has been suggested [9,10]. Nonetheless, the androgenic hypothesis is controversial, as other reports have described low levels of testosterone in COVID-19 intensive care patients. In addition, an association between alopecia and COVID-19 among male patients has been ruled out by other authors [11].

This exploratory study aims to evaluate the prevalence of Telogen effluvium (TE) in a sample of patients that were consecutively evaluated after recovery from COVID-19 pneumonia, and to identify clinical and immunologic parameters associated with TE.

## Materials and methods

### Study design

This is a single centre cross-sectional study carried out enrolling the patients consecutively admitted from June to November 2020 at the Outpatient Clinic of the Respiratory Department of the University Hospital of Padova, Italy, within three months from COVID-19 pneumonia recovery. Medical records related to the acute COVID-19 phase were checked for each patient, and clinical and laboratory data were collected and analysed. All the study participants signed a general consent for the use of their de-identified clinical data for research, analysis, and reporting; data were anonymized by assigning a de-identified patient code. Ethical approval was waived by the local Ethics Committee in view of the fact that the study involved procedures that are part of an internal hospital protocol approved by the Regional Health Authority. The study was carried out in accordance with the Declaration of Helsinki of 1975.

### Study assessment

Demographic and clinical data of enrolled patients were collected as well as data on patients’ hospitalizations and administered treatment. The inclusion criteria were: adults over 18 years of age, history of Covid19 infection. Exclusion criterion: not having given consent to the processing of personal data. Recorded data are available on repository doi:10.17632/w7dtshj7gk.1.

TE was defined as a scalp disorder characterized by thinning or shedding of hair greater than normal and/or generalized hair loss. TE data related to the 3 months following recovery from COVID-19 pneumonia. In order to evaluate the extent of TE, a visual analogic scale (VAS) was used for thick hair length (shoulder, mid-back) by dividing a bundle of hairs of each patient into nine piles of increasing hair amount (10, 50, 100, 200, 300, 400, 500, 600 and 700 hairs) as previously described by Martinez-Velasco MA et al. [12].

### Samples

We collected blood samples in order to analyse: complete blood count, blood coagulation panel including prothrombin time (PT), activated partial thromboplastin time ratio (APTT), fibrinogen and D-dimer, C-reactive protein (CRP), troponin I and immunological parameters (IgA, IgM and IgG1–4).

Western blots were performed for the measurement of IL-1α, IL-1β and IL-6 according to the method descripted by Belov L et al. [13].

## Statistical analysis

We compared patients’ demographic and clinical characteristics between groups using proportions (%) for categorical variables and medians (1st quartile [Q1]–3rd quartile [Q3]) for quantitative variables, and tested differences using the Fisher’s exact and Wilcoxon rank-sum tests, respectively. We assessed the association between sex and TE using odds ratios (OR) with 95% CI obtained fitting logistic regression models and adjusting for hospital admission (*vs*. home care) as a proxy indicator of COVID-19 severity. Moreover, we obtained sex-adjusted association estimates (ORs with 95% CIs) between each patients’ characteristic and the presence of TE using logistic regression models with TE as the dependent variable and the characteristic as the independent variable. We identified sex as the most important adjustment factor given its crucial role both in TE and COVID-19. We provided results from both a complete-case analysis (deleting missing data listwise) and an analysis after applying Multiple Imputation by Chained Equations (MICE) with 10 imputed datasets. We used STATA software, release version 16.1 (StataCorp, College Station, TX) and R version 4.0.3 (http://www.R-project.org/).

## Results

Overall, 104 patients were recruited; 80 of them (77%) had been hospitalized in the Respiratory Intensive Care Unit (RICU) of the University Hospital of Padova and the remaining ones had been treated at home. Men were more likely to be admitted to hospital compared to women (85 *vs.* 67%, respectively, *p* = .036) (Table 1). No other relevant differences were observed between males and females according to demographic, clinical and immunological data, except for a lower prevalence of dyspnoea among males (49 *vs.* 91% in females, *p* < .001) and a slightly higher count of platelets in females (median of 276 *vs*. 220 in males, *p* < .001).

**Table 1.** Distribution of demographic and clinical characteristics by sex^a^.

	Overall (*n* = 104)	Men (*n* = 59)	Women (*n* = 45)	*p* Value	Missing data (*n*)
Age (years)	63 (53–72)	66 (53–77)	58 (53–67)	.13	−
Current smoker (*n*; %)	37; 39	24; 42	13; 33	.40	8
BMI (kg/m^2^)	27 (24–30)	27 (25–30)	26 (23–29)	.36	−
Obesity (BMI >30 kg/m^2^) (*n*; %)	24; 23	15; 25	9; 20	.64	−
Number of comorbidities	3 (1–6)	3 (1–6)	3 (2–6)	.85	1
Hospital admission (*n*; %)	80; 77	50; 85	30; 67	.036	−
Length of stay in hospital^b^ (days)	20 (13–36)	21 (14–36)	19 (9–28)	.41	−
Remdesivir or Lopinavir^b^ (*n*; %)	43; 54	26; 52	17; 57	.82	−
Hyperimmune plasma and/or tocilizumab^b^ (*n*; %)	23; 29	18; 36	5; 17	.078	−
Methylprednisolone, enoxaparin, and/or hydroxychloroquine (*n*; %)	86; 83	51; 86	35; 78	.30	−
Amnesia (*n*; %)	25; 24	8; 14	17; 38	.005	−
Dyspnoea (*n*; %)	70; 67	29; 49	41; 91	<.001	−
Cough (*n*; %)	16; 16	6; 10	10; 24	.096	3
Interleukin 6 > 2 ng/l (*n*; %)	68; 79	42; 81	26; 76	.79	18
Interleukin 1 α > 2 ng/l (*n*; %)	13; 18	9; 20	4; 15	.75	33
Interleukin 1 β > 5 ng/l (*n*; %)	50; 68	32; 70	18; 64	.80	30
α_1_-antitrypsin g/l	1.3 (1.2–1.4)	1.3 (1.2–1.4)	1.3 (1.2–1.6)	.25	15
IgA g/l	1.9 (1.5–2.6)	1.9 (1.5–2.6)	1.9 (1.6–2.6)	.94	17
IgM g/l	0.9 (0.6–1.2)	0.8 (0.6–1.1)	1.1 (0.8–1.2)	.007	16
Total IgG g/l	10.7 (8.9–12.1)	10.5 (8.8–12.2)	10.9 (8.9–12.0)	.93	16
IgG1 g/l	7.0 (5.6–7.8)	7.1 (5.5–7.8)	7.0 (5.7–7.6)	.88	19
IgG2 g/l	2.6 (1.9–3.5)	2.8 (1.8–3.5)	2.6 (2.0–3.5)	.88	19
IgG3 g/l	0.3 (0.2–0.5)	0.3 (0.2–0.5)	0.3 (0.2–0.5)	.90	19
IgG4 g/l	0.5 (0.3–0.8)	0.6 (0.3–0.9)	0.5 (0.2–0.7)	.19	19
Leukocytes × 10^9/l	6 (5–8)	6 (5–8)	7 (6–8)	.34	8
Haemoglobin g/l	138 (129–146)	143 (130–152)	131 (124–139)	<.001	8
Platelets × 10^9/l	238 (204–290)	220 (191–251)	276 (233–313)	<.001	8
Neutrophils × 10^9/l	3 (3–5)	3 (3–5)	4 (3–5)	.41	8
Linfociti × 10^9/l	2 (2–3)	2 (2–2)	2 (1–3)	.80	8
D-dimer > 150 ug/l (*n*; %)	33; 37	19; 37	14; 38	1.00	15
CRp > 1.90 mg/l (*n*; %)	25; 26	14; 25	11; 28	.81	9
PT %	96 (88–107)	92 (86–104)	102 (93–110)	.007	20
APTTs	25 (24–27)	26 (24–28)	25 (24–26)	.17	21
Fibrinogen g/l	3 (3–3)	3 (2–3)	3 (3–3)	.38	15
Hs-Troponin > 2 ng/l (*n*; %)	44; 58	31; 72	13; 39	.005	28
BNP ng/l	25 (15–54)	25 (13–56)	24 (15–38)	.75	24
Albumin%	61 (58–63)	61 (59–63)	60 (57–63)	.33	21
α_1_-globulin%	4 (4–4)	4 (4–4)	4 (4–5)	.80	21
α_2_-globulin%	10 (9–11)	10 (8–11)	11 (9–11)	.18	21
β_1_-globulin%	6 (6–6)	6 (6–6)	6 (6–7)	.090	21
β_2_-globulin%	5 (4–6)	5 (4–6)	5 (4–6)	.85	21
γ-globulin%	14 (13–16)	14 (13–16)	14 (12–15)	.55	21

^a^*n* (%) or median (Q1–Q3) reported.

^b^Only assessed among the hospitalized patients.

Thirty-two patients (31%) reported a history of TE at the time of the examination and their mean TE VAS score was 5.78 ± 1.72 (range 3 − 9). Four of them underwent non-hormonal therapy, one subject used a topical treatment with minoxidil, and three subjects were administered oral vitamin 8 (biotin) supplement. Twenty-two patients (69%) had no longer signs of TE at the time of the visit. Women had about a 5-fold higher risk (odds) of TE compared to males (OR = 4.69, 95% CI: 1.91, 11.49; *p* = .001), and the association became stronger when adjusted for hospitalization (OR = 6.09, 95% CI: 2.34, 15.88; *p* < .001). Patients admitted to hospital had a 3-fold higher risk of TE compared to not hospitalized patients after taking sex into account (Table 2). Though hospitalized patients were mainly males (62%), only 18% of the hospitalized men complained of TE in comparison with 60% among hospitalized women.

**Table 2.** Distribution of demographic and clinical characteristics among the patients without and with Telogen effluvium (TE) and odds ratios adjusted for sex^a^.

	Without TE (*n* = 72)	With TE (*n* = 32)	Unadjusted *p* value	Adjusted OR, original dataset (95% CI)	Adjusted OR, imputed dataset (95% CI)^c^
Female sex (*n*; %)	23; 32	22; 69	<.001	–	–
Age (years)	62 (53–73)	65 (54–70)	.86	1.02 (0.98, 1.06)	–
Current smoker (*n*; %)	23; 35	14; 47	.37	2.12 (0.81, 5.77)	1.87 (0.70, 4.98)
BMI (kg/m^2^)	27 (24–29)	27 (25–31)	.79	1.01 (0.93, 1.10)	–
Obesity (BMI > 30 kg/m^2^) (*n*; %)	15; 21	9; 28	.45	1.81 (0.64,5.11)	–
Number of comorbidities	3 (1–6)	4 (2–7)	.32	1.11 (0.96, 1.30)	1.10 (0.95, 1.28)
Hospital admission (*n*; %)	53; 74	27; 84	.31	3.38 (1.09, 12.27)	–
Length of stay in hospital^b^ (days)	19 (13–28)	20 (10–56)	.57	1.02 (1.00, 1.04)	–
Remdesivir or Lopinavir^b^ (*n*; %)	26; 49	17; 63	.34	1.81 (0.63, 5.18)	–
Hyperimmune plasma and/or tocilizumab^b^ (*n*; %)	18; 34	5; 19	.20	0.63 (0.18, 2.13)	–
Methylprednisolone enoxaparin, and/or hydroxychloroquine (*n*; %)	55; 76	31; 97	.011	15.77 (2.77, 300.75)	–
Amnesia (*n*; %)	14; 19	11; 34	.14	1.43 (0.51, 3.91)	1.43 (0.52, 3.94)
Dyspnoea (*n*; %)	44; 61	26; 81	.069	1.42 (0.45, 4.67)	1.42 (0.45, 4.46)
Cough (*n*; %)	9; 13	7; 23	.24	1.43 (0.43, 4.63)	1.39 (0.43, 4.54)

^a^Median (Q1–Q3) is reported for quantitative variables.

^b^Only assessed among the hospitalized patients.

^c^Multiple imputation was conducted using all the variables listed in Tables 2 and 3, except for those only assessed among the hospitalized patients; association analyses were not conducted when the data were complete.

Moreover, our data showed a relationship between markers of COVID-19 severity and TE (Table 2). Of note, an association was also described with the presence of concomitant diseases (OR_adj_ 1.11, 95% CI 0.96, 1.30, *p* = .15, for each additional comorbidity), treatment with methylprednisolone, enoxaparin or hydroxychloroquine (OR_adj_ 15.77, 95% CI 2.77, 300.75, *p* = .011), and length of hospitalization (OR_adj_ 1.02, 95% CI 1.00, 1.04, *p* = .093, per a 1-d longer stay). Increased levels of inflammation parameters, such as CRP levels > 1.90 mg/l (OR_adj_: 2.43, 95% CI 0.85, 7.05, *p* = .096) or interleukin 1 β levels > 5 ng/l (OR_adj_ 4.72, 95% CI: 1.31, 23.19, *p* = .03) were also significantly associated with TE (Table 3). The results obtained after multiple imputation were consistent with the complete-case analysis (Tables 2 and 3).

**Table 3.** Distribution of biomarkers among the patients without and with Telogen effluvium (TE), and odds ratio adjusted for sex^a^.

	Without TE (*n* = 72)	With TE (*n* = 32)	Unadjusted *p* value	Adjusted OR, original dataset (95% CI)	Adjusted OR, imputed dataset (95% CI)^b^
Interleukin 6 > 2 ng/l (*n*; %)	49; 79	19; 79	.99	1.10 (0.34, 4.02)	0.76 (0.25, 2.32)
Interleukin 1 α > 2 ng/l (*n*; %)	10; 19	3; 16	.99	0.87 (0.17, 3.46)	1.20 (0.34, 4.24)
Interleukin 1 β > 5 ng/l (*n*; %)	32; 60	18; 86	.053	4.72 (1.31, 23.19)	3.59 (1.13, 11.4)
α_1_-antitrypsin g/l (*n*; %)	1.3 (1.2–1.4)	1.3 (1.2–1.6)	.22	4.27 (0.48, 42.88)	3.72 (0.43, 32.16)
IgA g/l (*n*; %)	2.0 (1.6–2.6)	1.8 (1.3–3.5)	.95	1.28 (0.80, 2.02)	1.24 (0.75, 2.02)
IgM g/l (*n*; %)	0.9 (0.6–1.2)	0.8 (0.4–1.1)	.60	0.42 (0.09, 1.04)	0.47 (0.12, 1.81)
Total IgG g/l (*n*; %)	10.5 (8.9–12.0)	11.0 (8.8–12.1)	.76	1.04 (0.86, 1.26)	1.04 (0.87, 1.25)
IgG1 g/l (*n*; %)	7.1 (5.6–7.8)	6.9 (5.5–7.5)	.90	1.12 (0.83, 1.51)	1.18 (0.90, 1.55)
IgG2 g/l (*n*; %)	2.7 (1.8–3.6)	2.3 (1.9–3.5)	.47	0.81 (0.51, 1.27)	0.86 (0.55, 1.35)
IgG3 g/l (*n*; %)	0.3 (0.2–0.4)	0.4 (0.2–0.6)	.18	6.43 (0.86, 54.11)	6.30 (0.73, 54.73)
IgG4 g/l (*n*; %)	0.6 (0.3–0.9)	0.5 (0.2–0.6)	.30	0.78 (0.22, 2.34)	0.77 (0.26, 2.30)
Leukocytes × 10^9/l	6 (5–8)	7 (6–8)	.47	0.95 (0.78, 1.14)	0.97 (0.80, 1.17)
Haemoglobin g/l	139 (129–149)	131 (128–140)	.048	0.99 (0.95, 1.02)	0.99 (0.95, 1.02)
Platelets × 10^9/l	231 (201–266)	276 (229–323)	.010	1.01 (1.00, 1.01)	1.01 (1.00, 1.01)
Neutrophils × 10^9/l	3 (3–5)	4 (3–5)	.45	0.96 (0.74, 1.22)	0.99 (0.78, 1.26)
Linfociti × 10^9/l	2 (2–2)	2 (2–3)	.88	0.91 (0.55, 1.33)	0.93 (0.61, 1.43)
D-dimer > 150 ug/l (*n*; %)	24; 39	9; 33	.81	0.75 (0.27, 2.03)	0.82 (0.30, 2.22)
CRp > 1.90 mg/l (*n*; %)	14; 21	11; 38	.13	2.43 (0.85, 7.05)	2.23 (0.79, 6.30)
PT%	94 (88–104)	103 (95–110)	.036	1.01 (0.98, 1.05)	1.01 (0.98, 1.04)
APTTs	25 (24–27)	25 (24–27)	.83	1.08 (0.89, 1.33)	1.09 (0.89, 1.33)
Fibrinogen g/l	3 (3–3)	3 (3–3)	.86	0.87 (0.42, 1.71)	0.84 (0.43, 1.64)
Hs-Troponin > 2 ng/l (*n*; %)	30; 58	14; 58	1.00	1.85 (0.61, 6.10)	1.65 (0.53, 5.12)
BNP ng/l	22 (12–48)	35 (23–69)	.050	1.00 (1.00, 1.01)	1.00 (0.99, 1.01)
Albumin%	61 (58–63)	62 (57–63)	.68	1.05 (0.90, 1.23)	1.03 (0.88, 1.2)
α_1_-globulin%	4 (3–4)	4 (4–5)	.27	1.36 (0.74, 2.63)	1.30 (0.73, 2.32)
α_2_-globulin%	10 (9–11)	10 (9–11)	.79	0.93 (0.71, 1.20)	0.94 (0.72, 1.22)
β_1_-globulin%	6 (6–6)	6 (6–7)	.38	0.96 (0.45, 1.83)	0.95 (0.46, 1.95)
β_2_-globulin%	5 (4–6)	5 (5–6)	.24	1.42 (0.85, 2.42)	1.32 (0.80, 2.19)
γ-globulin%	14 (13–16)	14 (11–15)	.31	0.88 (0.71, 1.09)	0.92 (0.75, 1.12)

^a^Median (Q1–Q3) is reported for quantitative variables.

^b^Multiple imputation was conducted using all the variables listed in Tables 2 and 3, except for those only assessed among the hospitalized patients.

## Discussion

Hair loss is one of the under-evaluated sequelae of COVID-19 disease. Those who were infected with the virus were under immense psychosocial and physiologic stress [14]. Though hair loss is considered a minor clinical problem, it is responsible for a not negligible emotional impact, it affects patients’ quality of life and it increases the risk of anxiety and depression [15,16]. As reported by other Authors diffuse hair shedding occurs 2 − 3 months after COVID-19 infection [17,18] and the progression of the disease is related to an increased level of stress [19], which is higher in women [20]. In line with our findings, a higher prevalence of androgenic alopecia in males [21] had been previously reported, as well as a higher risk of severe COVID-19 disease for males compared to females [1,2]. This gender gap is probably associated with the up-regulation of TMPRRS2 and the ACE2 receptor, which are mainly involved in the coronavirus infection. Accordingly, other conditions driven by androgens, including androgenic alopecia, baldness or the presence of grey hair were more commonly observed in males with COVID-19 disease. Differently from the above-mentioned reports [4,21], in our study the presence of TE was significantly more common in women, in accordance with the report by Xiong et al. [11], describing alopecia as a sequela of COVID-19 disease exclusively in females. We also observed that a higher severity of the disease plays a more important role than hormonal influence, in agreement with the results of the study of Salazar Arenas MÁ et al. [22]. Of note, in the studies showing a higher prevalence of alopecia in males [4,21,23], patients were not stratified according to the severity of the disease; in addition, other studies reported low levels of androgens in patients admitted to an intensive care unit [24,25]. These findings point to a potential therapeutic role of finasteride or other five alpha inhibitors in COVID-19 patients based on the hormonal hypothesis. However, the trade-off with the potential side effects of this class of drugs should be carefully evaluated. Indeed, hair loss may represent the side effects of several treatments currently used in COVID-19 therapy, such as antiviral, antimalarial or immunosuppressive agents, which are being more aggressively used in more severe patients [26]. The severity of inflammation could also play a role as suggested by the higher levels of CRP and platelets [27] among patients with alopecia. Furthermore, in our study patients with TE had higher levels of interleukin 1 β, which has been demonstrated to be a potent inhibitor of hair growth *in vitro* [28]. Though the role of this cytokine in hair loss among patients with previous COVID-19 disease is still under evaluation, we highlight that higher concentrations of interleukin 1 β have been detected in alopecia areata, which is for some aspects related to TE. The development of TE is still unpredictable and characterized by relapses which are often more serious than the primary episode [29]. The limit of the study is the small number of the sample, however, in the statistical analysis, we applied a model that considers this potential bias, as well as confounding factors, such as sex and smoking. Further studies will be needed to understand the long-term prevalence and prognosis of TE associated with COVID-19 infection. Instead the strengths of the study are that it included a control group, the recording of comorbidities and all the drugs taken Furthermore, compared to other studies [17,18] the population is representative of the general population and it was not selected at a dermatological clinic.

## Conclusion

Hair shedding has been frequently observed in patients with previous COVID-19 disease. Although it is plausible that androgens have an aetiologic role, this exploratory study raises the hypothesis that hair shedding is more strictly related to the severity of COVID-19 disease and the underlying inflammation rather than to patients’ hormonal status. In particular, a role for the pro-inflammatory cytokine interleukin 1 β can be hypothesized. Long-term studies are needed to evaluate the pathogenesis and reversibility of TE among patients with a previous diagnosis of COVID-19 infection.

## Data Availability

The data is available for reproduction of results on request from the corresponding author.
